# Effects of perceived teacher support on motivation and engagement amongst Chinese college students: Need satisfaction as the mediator

**DOI:** 10.3389/fpsyg.2022.949495

**Published:** 2022-08-25

**Authors:** Lihua Zhou, Yabing Gao, Jiangbo Hu, Xiaoqing Tu, Xiaoxian Zhang

**Affiliations:** ^1^School of Education, Zhejiang International Studies University, Hangzhou, China; ^2^Institute for Studies on Education Governance, Zhejiang International Studies University, Hangzhou, China; ^3^Institute for China-South Africa Comparative Education Studies, Zhejiang International Studies University, Hangzhou, China; ^4^Faculty of Arts, Macquarie School of Education, Macquarie University, Sydney, NSW, Australia; ^5^Jing Hengyi School of Education, Hangzhou Normal University, Hangzhou, China

**Keywords:** teacher support, motivation, class engagement, need satisfaction, self-determination theory, Chinese college students

## Abstract

College students' motivation and engagement are regarded as essential factors to promote their academic development and wellbeing. However, motivation and engagement among college students appear to decline after they enter the university. Guided by the framework of self-determination theory, this study attempted to explore a motivational model of how three dimensions of perceived teacher support (autonomy, structure, and involvement) related to student motivation and class engagement, using need satisfaction as a mediator. Drew on a survey of the perceptions of 705 Chinese university students, the results showed that besides structure, both autonomy support and involvement positively related to students' need satisfaction. Further, need satisfaction was positively associated with autonomous motivation, controlled motivation, and class engagement and negatively linked with amotivation. Yet, only autonomous motivation was positively predicted for class engagement. Need satisfaction and the chain from need satisfaction to autonomous motivation were found to be the significant mediators. The practical implications of educational practices are discussed.

## Introduction

College students' motivation and engagement have a predictive effect on their school success and adaptive development, such as academic performance (Taylor et al., 2014) and subjective wellbeing (Hope et al., 2019). Nonetheless, evidence shows that college students' motivation and class engagement tend to decline after they enter the university (Trolian and Jach, 2020). Autonomous motivation among Chinese college students has been reported to fall rapidly from year one to year two (Pan and Gauvain, 2012). Hence, it is imperative to identify the predictors for promoting students' motivation and engagement in their college study, especially at the commencement of college, which is the current study's primary objective.

This study draws on self-determination theory (SDT; Deci and Ryan, 2000; Ryan and Deci, 2020) to explore the core factors associated with students' motivation and engagement. As a well-established theory, SDT provides a prominent framework that differentiates between quality and quantity of motivation, and also postulates a model to explain how contextual (e.g., perceived teacher support) and personal (i.e., need satisfaction) factors can jointly contribute to student motivation and engagement. Nevertheless, only few studies applied SDT to examine this full motivational sequence in a single integrated model (e.g., Zhou et al., 2019b; Leo et al., 2022). Grounded on SDT, the present study aims to test how Chinese college students' perceptions of their teachers' support for autonomy, structure, and involvement relate to their need satisfaction and consequently their different types of motivation, which in turn, link to their class engagement.

## SDT-based motivational research

### Student motivation and class engagement

Students participate in school activities for different reasons (Deci and Ryan, 2000; Guay, 2021). According to the extent of self-determination among these reasons, SDT has distinguished three distinctive but continuous types of motivation, namely intrinsic motivation, extrinsic motivation, and amotivation (Deci and Ryan, 2000; Ryan and Deci, 2020). *Intrinsic motivation* refers to engaging in a learning activity for the sake of curiosity, interest, or enjoyment. It represents the highest level of autonomy or self-determination. *Extrinsic motivation* pertains performing behaviors for instrumental reasons to achieve other benefits, which includes four extrinsic motivation subtypes, namely, external, introjected, identified, and integrated regulations (from low to high level of self-determination) (Deci and Ryan, 2000). In schooling life, external regulation occurs when a student is driven to academic activities by external pressures (e.g., rewards or punishments). Introjected regulation relates to being motivated to learning activities by internal pressures (to obtain self-esteem or to avoid feeling guilt or shame). Identified regulation is exhibited when students are driven to study because they find its value and significance. Integrated regulation occurs when a learning behavior is consistent with students' sense of self. Finally, *amotivation* refers to the absence of any intention and volition to involve in a learning activity (Ryan and Deci, 2020).

Recent meta-analyses have proven the strong intercorrelations among identified regulation, integrated regulation, and intrinsic motivation (Vasconcellos et al., 2020). These three forms of motivation have been combined into autonomous motivation, representing high-quality motivation, whereas external and introjected regulations have typically been conceptualized as controlled motivation, reflecting low-quality motivation (Deci and Ryan, 2000). Indeed, autonomous motivation, controlled motivation, and amotivation have been widely discussed in SDT literature (Vasconcellos et al., 2020; Guay, 2021; Leo et al., 2022). Research has indicated that integrated regulation and intrinsic motivation share conceptual properties, which cause difficulties to differentiate between these two motivation subtypes (Vasconcellos et al., 2020; Bureau et al., 2022). Consequently, integrated regulation is rarely assessed in SDT studies on students whose identity is still developing (Guay, 2021; Bureau et al., 2022). Following prior SDT research (e.g., Amoura et al., 2015; Leo et al., 2022), the current study examined Chinese college students' autonomous motivation, controlled motivation, and amotivation, yet did not include their integrated regulation.

Numerous SDT-based studies have revealed that academic motivation is associated with class engagement, which has been described as behavioral, emotional, and cognitive involvement in the classroom (Fredricks et al., 2004). Autonomous motivation has been found to positively predict engagement across various domains like one specific subject (mainly in physical education, PE; Leo et al., 2022), general classes (e.g., Zhou et al., 2019b), and competitive sports (Pelletier et al., 2001). This effect exists among schooling years from primary schools (e.g., Zhou et al., 2019b), high schools (e.g., Standage et al., 2005), to universities (e.g., Behzadnia et al., 2018). In contrast, controlled motivation and amotivation have been found to negatively predict engagement-related variables (e.g., Sánchez-Oliva et al., 2014). In PE classes, however, controlled motivation has been shown to positively link to high school students' concentration (Maldonado et al., 2019); or it is unable to statistically predict engagement-related variables (Behzadnia et al., 2018; Zamarripa et al., 2021; Leo et al., 2022). Also, amotivation has been found to fail to predict desirable outcomes (e.g., emotional engagement in PE; Standage et al., 2005). These contradicting findings suggest that diverse antecedents may affect students' motivation and engagement, and further investigation is needed for clarifying the associations amongst these variables.

### The importance of need satisfaction and need support

SDT proposes that one personal factor, specifically, the satisfaction of three basic needs for autonomy, competence, and relatedness, can foster students' psychological growth (e.g., motivation and engagement), regardless of students' cultural background (Deci and Ryan, 2000; Ryan and Deci, 2020). *The need for autonomy* is conceptualized as a general feeling of willingness and freedom to perform an activity. *The need for competence* reflects the desire to feel effectiveness, mastery, accomplishment, and achievement. *The need for relatedness* corresponds to the sense of being accepted by significant others, belonging to a group, and connecting with the social world (Ryan and Deci, 2020). Considerable SDT-based research has demonstrated that, within the school context, students who experience high levels of need satisfaction can predict more high-quality motivation and class engagement (e.g., Zhou et al., 2019b). Nevertheless, the pattern of associations between need satisfaction and low-quality motivation was reported differently in the literature (e.g., Vasconcellos et al., 2020). Some scholars identified that the association between need satisfaction and controlled motivation is positive (e.g., Sánchez-Oliva et al., 2014), though some found it is non-significant (e.g., Behzadnia et al., 2018). Inconsistent with SDT, elementary school students' amotivation in PE was not predicted by their need satisfaction (e.g., Sánchez-Oliva et al., 2014). Therefore, further research is required to identify the relations between need satisfaction and low-quality motivation (controlled motivation and amotivation) within SDT sequential model.

As one important social agent in school, teachers can nurture students' three fundamental needs through their teaching practices (Deci and Ryan, 2000; Ryan and Deci, 2020). Within SDT, *teacher support* refers to those supportive teaching behaviors that can satisfy students' basic psychological needs, which includes three interrelated but independent dimensions, namely autonomy support, structure, and involvement (Reeve et al., 2004; Ryan and Deci, 2020). *Autonomy support* points to the teaching behaviors inspiring students' inner motivational resource, which consists of supporting students' interests and preferences, adopting students' perspectives, giving rationales for mandatory learning tasks, encouraging students to make their own decisions, and accepting students' negative emotions. *Structure*, which is seen as competence support, is described as all those teaching strategies to provide a predictable and consistent learning environment, mainly being accomplished by explaining realistic goals and expectations, giving clear instructions, offering appropriate feedback, and providing optimal challenges. Finally, *involvement*, namely relatedness support, is related to students' feeling to bond with their teachers, which is usually practiced by teachers displaying affection toward students, providing inspiration and appreciation, dedicating time and resources, and communicating sympathetically (Reeve et al., 2004; Ryan and Deci, 2020).

Although SDT posits all three dimensions of teacher support that are indispensable for student motivation and engagement, much of the empirical research has solely confirmed the unique effects of overall teacher support (e.g., Leo et al., 2022) or one dimension of teacher support (mainly in autonomy support) (e.g., Zhou et al., 2019b). Only limited SDT research has simultaneously explored the joint effects of all three aspects of teacher support on students' motivational outcomes (Stroet et al., 2013; Hornstra et al., 2021; Olivier et al., 2021). On the one hand, some research has found that all three teacher support dimensions relate positively to motivational outcomes, such as, need satisfaction and motivation in PE among British students with an age mean of 12.81 years (Taylor and Ntoumanis, 2007), reading motivation among Flemish students of 15 years old (De Naeghel et al., 2014), as well as need satisfaction and class engagement among American students in 1st through 12th grade (Tucker et al., 2002). However, conversely, other research has shown that not each of the three teacher support aspects can predict motivational outcomes. For instance, only students' perceived structure and involvement, but not autonomy support, could predict their engagement (Skinner and Belmont, 1993). Except for the positive effects of autonomy support and involvement, teacher-reported structure negatively predicted students' assessed need satisfaction in school (Hornstra et al., 2020). Observed teachers' structure before PE activity could negatively predict students' engagement (González-Peño et al., 2021); whereas students' reported structure in a Dutch language class failed to predict student engagement being measured by student, teacher, and observer (Lietaert et al., 2015). Thus, the current study would assist to clarify the combined contributions of perceived teachers' autonomy support, structure, and involvement to the motivational process of students.

## The present study

Overall, the aforementioned findings have supported the SDT-based motivational model of teacher support → need satisfaction → motivation → engagement (Vallerand, 1997; Ryan and Deci, 2020). However, several prior work features limit the possibility to draw a definitive conclusion. Our work contributes to this research field in the following aspects.

Firstly, the bulk of SDT research has focused on autonomy support or considered teacher support as one overall factor. Very little attention has been devoted to the combined roles of autonomy support, structure, and involvement (Hornstra et al., 2021; Olivier et al., 2021). It is recognized, however, that autonomy support, structure, and involvement all should be considered to facilitate motivational processes (Ryan and Deci, 2020). Hence, the present study was designed to identify the role of the three aspects of teacher support, which would extend previous findings.

Secondly, numerous SDT studies have examined the interrelations among several motivational variables, yet only few studies have tested the full sequence of associations within one single integrated model (Leo et al., 2022). So far, the chain of need satisfaction to autonomous motivation has been revealed to mediate the association between overall teacher support and engagement (Standage et al., 2005; Zhang et al., 2011), as well as between teacher autonomy support and engagement-related variables (Leo et al., 2022). As discussed earlier, however, the relations between need satisfaction and low-quality motivation (controlled motivation and amotivation) as well as between the latter and student engagement have been demonstrated to be mixed and inconclusive (Standage et al., 2005; Sánchez-Oliva et al., 2014; Behzadnia et al., 2018; Leo et al., 2022). As such, the application of the full SDT-sequential model would help to clarify the possible mediating role of need satisfaction and different motivation types in the relations between the three aspects of teacher support and students' class engagement.

Thirdly, accumulated evidence in this area has mainly been based on students from Western individualistic contexts (e.g., Standage et al., 2005; Zhang et al., 2011; Leo et al., 2022). Some Chinese researchers questioned that, under the collectivistic context, autonomy appears to be unlikely to contribute to optimal outcomes (e.g., Wu et al., 2014). Consequently, it is worthwhile to investigate the SDT-sequential model according to a sample from China, which is representative of the typically Eastern collectivistic culture (Zhou et al., 2019a). Further, as mentioned above, autonomous motivation was found to dramatically decline between freshmen and sophomores in China. Therefore, we attempted to explore the prerequisite of motivation and engagement among Chinese university students, which has rarely been investigated in SDT research.

Finally, most of the earlier studies have predominantly been conducted only on one subject (mainly in PE) (Vasconcellos et al., 2020). Nevertheless, students can be affected by the teachers in different subjects rather than teachers of one subject. While motivation in one specific subject is more likely to be supported by the teacher who is teaching this subject, motivation in the general learning activities is more likely to be personality-driven (Vallerand, 1997). It is necessary to test the predictive role of need support provided by all of the involved teachers.

In summary, to our knowledge, no existing literature has examined the full SDT-sequential model that simultaneously considers the joint effect of perceived teachers' autonomy support, structure, and involvement on three motivation subtypes and class engagement among Chinese college students. Specifically, grounded on SDT and the past findings, we formulated the following hypotheses: (1) Perceived teachers' autonomy support (Hypothesis 1a), structure (Hypothesis 1b), and involvement (Hypothesis 1c) would all positively relate to students' overall need satisfaction; (2) The latter would be positively associated with autonomous motivation (Hypothesis 2a), controlled motivation (Hypothesis 2b), and class engagement (Hypothesis 2c), whereas negatively related with amotivation (Hypothesis 2d); (3) Class engagement would be predicted by autonomous motivation (positively, Hypothesis 3a), controlled motivation (negatively, Hypothesis 3b), and amotivation (negatively, Hypothesis 3c), irrespectively; (4) The associations between three teacher support dimensions and engagement could be mediated by need satisfaction (Hypothesis 4a) and the chains from need satisfaction to autonomous motivation (Hypothesis 4b), controlled motivation (Hypothesis 4c), and amotivation (Hypothesis 4d).

## Materials and methods

### Participants and procedure

By randomized cluster sampling method, 705 students from 22 classrooms were recruited from a university located in southeast China. Of this sample, the mean age was 18.45 years (*SD* = 0.66, ranging = 17–23 years). The majority of the students were female (79.72%) and freshmen (70.5%). With regards to their major, the students studying human sciences (e.g., English, music, and economy) and natural sciences (e.g., maths, physics, and computer science) accounted for 53.9 and 46.1%, respectively (see Table 1).

**Table 1:** Basic characteristics of participants.

**Variables**		***n* (%)**
Age	Mean ± SD	18.45 ± 0.66
Gender	Male	143 (20.28%)
	Female	562 (79.72%)
Grade	Freshman	497 (70.50%)
	Sophomore	208 (29.50%)
Birthplace	Rural areas	490 (69.50%)
	Urban areas	215 (30.50%)
Major	Human sciences	380 (53.90%)
	Natural sciences	325 (46.10%)

The ethical approval was granted by the University of the first author. Consent forms were collected from the participating students who read the information letter that introduced the research purpose, the process of data collection, and the instructions regarding the anonymous, confidential, and voluntary nature of the study. The participating students were told that there were no right or wrong answers, and they were encouraged to respond to each item honestly according to their own experiences. To minimize the honeymoon bias, the questionnaires were administered at the end of the semester after students had enough communication with their teachers. Data were collected in the classroom. Students spent ~15 min filling out the questionnaires voluntarily without compensation.

### Measures

The main variables of the study including perceived teacher support, need satisfaction, motivation, and class engagement were measured with specific tools. Students rated each item on a five-point Likert scale from 1 (strongly disagree) to 5 (strongly agree). Each variable score was calculated by averaging its respective item scores.

#### Perceived teacher support

To tap students' perceptions of teacher support, we used the shortened version of the Teacher as Social Context scale (TASC; Belmont et al., 1992; Haerens et al., 2013). Each dimension (autonomy support, structure, and involvement) consists of six items. This tool has been proved to be reliable and valid among Chinese students (Zhou, 2016).

In the current study, two items from the autonomy subscale (“My teachers don't explain why what I do in school is important to me”) and from the involvement subscale (“I can't depend on my teachers for important things.”) reduced the internal consistency of its subscale, thus, these two items were removed. Finally, 16 items were used to assess the perceived autonomy support (five items, e.g., “My teachers listen to my ideas.”), structure (five items, e.g., “Teachers show me how to solve problems for myself”), and involvement (six items, e.g., “My teachers like me”), respectively.

#### Need satisfaction

We used the Activity-Feelings States (AFS; Reeve and Sickenius, 1994) to evaluate the levels of satisfaction of three basic psychological needs. The stem for this scale was “During classes, I feel…”. Each subscale consists of three items: autonomy need (e.g., “freedom”), competence need (e.g., “capable”), and relatedness need (e.g., “I belong and the people here care about me”). All items were averaged to represent overall need satisfaction. Previous research has demonstrated the AFS to be reliable and valid in assessing need satisfaction among students in China (e.g., Zhou et al., 2017, 2019b) and other Asian countries (e.g., Japan, Oga-Baldwin et al., 2017).

##### Motivation

To clarify students' motivation for attending college, the Academic Motivation Scale (AMS; Vallerand et al., 1992) with 28 items was used. Participants responded to the items following the stem, “Why do you go to college?” Autonomous motivation was assessed by 16 items, including 12 items from intrinsic motivation (e.g., “Because for me, college is fun”) and four items from identified regulation (e.g., “Because I think that education will help me better prepare for the career that I have chosen”). Controlled motivation was evaluated by eight items, concerning four items each from external regulation (e.g., “In order to obtain a more prestigious job later on”) and introjected regulation (e.g., “To show myself that I am an intelligent person”). Finally, amotivation consisted of four items (e.g., “Honestly, I don't know, I really feel that I am wasting my time in college”). The reliability and validity of AMS have been demonstrated by the work of Chen (2007) among Chinese participants.

#### Class engagement

To capture the general perceptions of class engagement, the Individual Self-Report Engagement scale was used (Jang et al., 2010; Zhou et al., 2019b). The stem for the scale was “During classes, I…”. The questionnaire included behavioral engagement (two items; e.g., “paid attention”), cognitive engagement (one item; “tried to learn as much as I could”), and emotional engagement (one item; “enjoyed the lessons”).

### Data analysis

The preliminary results were calculated in SPSS Statistics 27.0. Research has shown that some socio-demographic factors are associated with motivational variables, such as gender, grade, family site, and college major (e.g., De Naeghel et al., 2014; Maulana et al., 2016; Vasconcellos et al., 2020; Opdenakker, 2021). Accordingly, we tested the bivariate relations between these four socio-demographic factors and the substantive variables. To assure the models' parsimoniousness, only significant socio-demographic factors were included as covariates for the main analyses. Additionally, we also tested the risk of multicollinearity among autonomy support, structure, and involvement. The results of collinearity statistics suggested no potential multicollinearity (tolerance = 0.651 > 0.50; variation inflation factor [VIF] = 1.535 < 2.0).

The main analyses were performed by Mplus Version 8.3 (Muthén and Muthén, 1998-2018). Due to the nested data (i.e., the students belonging to 22 classrooms), we calculated the intra-class correlations (ICCs). Except for autonomy support and amotivation, the results of ICCs were lower than 0.10 (see Table 2), demonstrating that the substantial variance was at the student level. Further, the number of classrooms (*n* = 22) was not sufficient to test the proposed model at the classroom level. Hence, to account for the nonindependence of observations of the nesting data, we set “classroom” as the clustering variable through the “Type = Complex” option in Mplus. Meanwhile, apart from the dependent variable (i.e., class engagement), all study variables were group-mean centered around the classroom mean.

**Table 2:** Descriptive statistics, ICC, and correlations among the study variables.

	**1**	**2**	**3**	**4**	**5**	**6**	**7**	**8**	**9**	**10**	**11**	**12**
1. Gender	1											
2. Grade	−0.08^*^	1										
3. Family Site	0.05	0.02	1									
4. Major	−0.14^**^	0.68^**^	−0.04	1								
5. Autonomy Support	0.09^*^	−0.08^*^	0.12^**^	−0.12^**^	1							
6. Involvement	0.03	−0.01	0.15^**^	−0.06	0.62^**^	1						
7. Structure	0.06	0.02	0.17^**^	−0.05	0.66^**^	0.58^**^	1					
8. Overall Need Satisfaction	0.08^*^	−0.04	0.05	−0.06	0.44^**^	0.50^**^	0.38^**^	1				
9. Autonomous Motivation	0.18^**^	−0.11^**^	0.09^*^	−0.17^**^	0.35^**^	0.38^**^	0.29^**^	0.51^**^	1			
10. Controlled Motivation	0.12^**^	0.10^**^	0.08^*^	0.02	0.14^**^	0.20^**^	0.19^**^	0.30^**^	0.50^**^	1		
11. Amotivation	−0.15^**^	0.21^**^	−0.11^**^	0.19^**^	−0.31^**^	−0.30^**^	−0.25^**^	−0.34^**^	−0.43^**^	−0.06	1	
12. Class Engagement	0.18^**^	−0.03	0.08^*^	−0.10^**^	0.39^**^	0.48^**^	0.33^**^	0.55^**^	0.48^**^	0.23^**^	−0.36^**^	1
*Mean*					3.16	2.94	2.93	3.47	3.79	3.54	2.21	3.26
*SD*					0.61	0.66	0.60	0.57	0.54	0.58	0.74	0.66
α					0.74	0.83	0.72	0.82	0.86	0.75	0.70	0.80
ω					0.74	0.83	0.70	0.82	0.86	0.75	0.70	0.79
*ICC*					0.14	0.07	0.09	0.02	0.06	0.03	0.11	0.06

N = 705; α, Cronbach's alpha; ω, omega value; ICC, Intraclass Correlation Coefficient.

* p < 0.05,

** p < 0.01, the same as below.

Structural equation modeling analysis (SEM) was conducted to verify the proposed models. So far, the estimation of sample size for SEM is flexible, and there is no rule of thumb that can be applied to all studies (Kyriazos, 2018). In this study, the ratio of the sample size to parameters is 5.83 (with 705 cases for 121 free parameters), which is below the strict ratio recommendation of 20 (Kline, 2016), but still in accord with the minimal five cases per parameter (Bollen, 1989). Due to the sample size, we used the parceling strategy, which was recommended in previous research (Little et al., 2002; Kline, 2016; Zhou et al., 2019b). According to the values of loading items, the latent variables for autonomy support, structure, and involvement were indicated by three parcels each, and autonomous motivation and controlled motivation were indicated by four parcels each. Parceling items are available upon request. Furthermore, the latent construct for overall need satisfaction was represented by its three subscales. In the hypothesized model, amotivation and class engagement were indicated by their four items, independently.

A two-step approach was used to test the presumed model (Anderson and Gerbing, 1988). Firstly, confirmatory factor analysis (CFA) was used to evaluate the measurement model. The proposed measurement model was an eight-factor model consisting of three teacher support dimensions, overall need satisfaction, three motivation subtypes, and class engagement; all eight factors were allowed to correlate with one another. In addition, we also tested a six-factor model which combined nine parcels of three teacher support dimensions into one factor, the rest was the same as the eight-factor model. Secondly, maximum likelihood estimation with robust standard errors (MLR) was conducted to examine the adequacy of the hypothesized model (Model 1). Meanwhile, the “IND” command in Mplus was used to compute the indirect effects. Model 1 was a full mediation model, that is, after controlling for significant demographic covariates, overall need satisfaction, followed by three motivation subtypes, would mediate the links between three teacher support dimensions and class engagement; and the three dimensions of teacher support were intercorrelated with each other, so did the three types of motivation.

Finally, according to SDT and past research, the alternative models were tested.^1^ As social support was indicated to link directly to motivation and engagement (Maulana et al., 2016; Olivier et al., 2021; Opdenakker, 2021), we examined a partial mediation model (Model 2). In Model 2, the three teacher support dimensions would directly predict three motivation types and class engagement, and the remaining was the same as Model 1. Moreover, both external regulation and introjected regulation have been found to uniquely contribute to engagement (e.g., Pelletier et al., 2001), hence, we tested Model 3. Model 3 included four motivation subtypes (autonomous motivation, introjected regulation, external regulation, and amotivation), and the rest was the same as Model 1.

Considering the sample size, an acceptable model fit is indicated by CFI and TLI values equal to or exceeding 0.90 as well as SRMR and RMSEA values lower than 0.08 (Schumacker and Lomax, 1996; Hu and Bentler, 1999; Van de Schoot et al., 2012). Further, a change in CFI values (ΔCFI > 0.01) is considered a significant difference between two nested models (Cheung and Rensvold, 2002).

## Results

### Preliminary analyses

Table 2 presents the Cronbach's alpha and omega values (ranging from 0.70 to 0.86) for each variable under study, indicating an acceptable internal consistency of each scale (Dunn et al., 2014). We also reported the CFA results of the TASC [χ^2^ (98) = 439.91, *p* < 0.001, RMSEA and 90% CI = 0.070(0.064–0.077), SRMR = 0.05, CFI = 0.92, TLI = 0.90], AFS [χ^2^ (20) = 68.90, *p* < 0.001, RMSEA and 90% CI = 0.059(0.044–0.074), SRMR= 0.03, CFI = 0.97, TLI = 0.95], and AMS [χ^2^ (327) = 1222.50, *p* < 0.001, RMSEA and 90% CI = 0.062(0.059–0.066), SRMR = 0.07, CFI = 0.86, TLI = 0.83], which supported the factorial structure of these scales.

As can be seen in Table 2, all three teacher support dimensions related positively to need satisfaction, autonomous and controlled motivation, and class engagement, but they were all negatively linked to amotivation. Next, need satisfaction was positively related to autonomous motivation, controlled motivation, and class engagement. Lastly, class engagement was positively associated with autonomous and controlled motivation. In general, these findings supported the expected associations among the study variables.

Table 2 also displayed the results of the correlations between the demographic factors and the study variables. We included the significant demographic factors in the models by adding them as predictors of each related latent variable. In addition, we looked at the correlations between each teacher support dimension and each need satisfaction aspect. The values of these interrelations (available upon request) were similar to each other, indicating that the three need satisfaction dimensions could be combined into one overall score to improve model parsimony.

To test the mean differences among the three dimensions of teacher support, we conducted the general linear model repeated measures test [Wilks' Lambda = 0.78, *F* (2, 703) = 98.06, *p* < 0.001, partial η^2^ = 0.22]. Follow up analyses suggested that students reported a higher level of teacher autonomy support, compared to structure [*p* < 0.001] and involvement [*p* < 0.001].

### Primary analyses

The CFA results were displayed in Table 3. Compared to the six-factor model, the eight-factor model was supported (ΔCFI = 0.037 > 0.01). Meanwhile, each observed variable in the eight-factor model strongly loaded on its corresponding latent factor (mean λ = 0.698).

**Table 3:** Fit statistics for the models.

**Model tested**	**χ^2^**	***df***	**χ^2^*/df***	***p***	**RMSEA and 90% CI**	**SRMR**	**TLI**	**CFI**
**CFA models**								
Six-factor model	1269.30	335	30.79	<0.001	0.063(0.059–0.067)	0.07	0.86	0.879
Eight-factor model	972.91	322	30.02	<0.001	0.054(0.050–0.057)	0.07	0.90	0.916
**SEM models**								
Model 1	1008.61	425	20.37	<0.001	0.044(0.041–0.048)	0.05	0.90	0.912
Model 2	979.233	413	20.37	<0.001	0.044(0.041–0.048)	0.05	0.90	0.915
Model 3	3514.44	548	60.41	<0.001	0.088(0.085–0.090)	0.07	0.60	0.645

Furthermore, SEM results showed that both Model 1 and Model 2 yielded acceptable fit indices, except for Model 3 (see Table 3). Compared to the full mediation model (Model 1), however, the partial mediation model (Model 2) did not show a better model fit (ΔCFI = 0.003 < 0.01).

**Figure 1:**
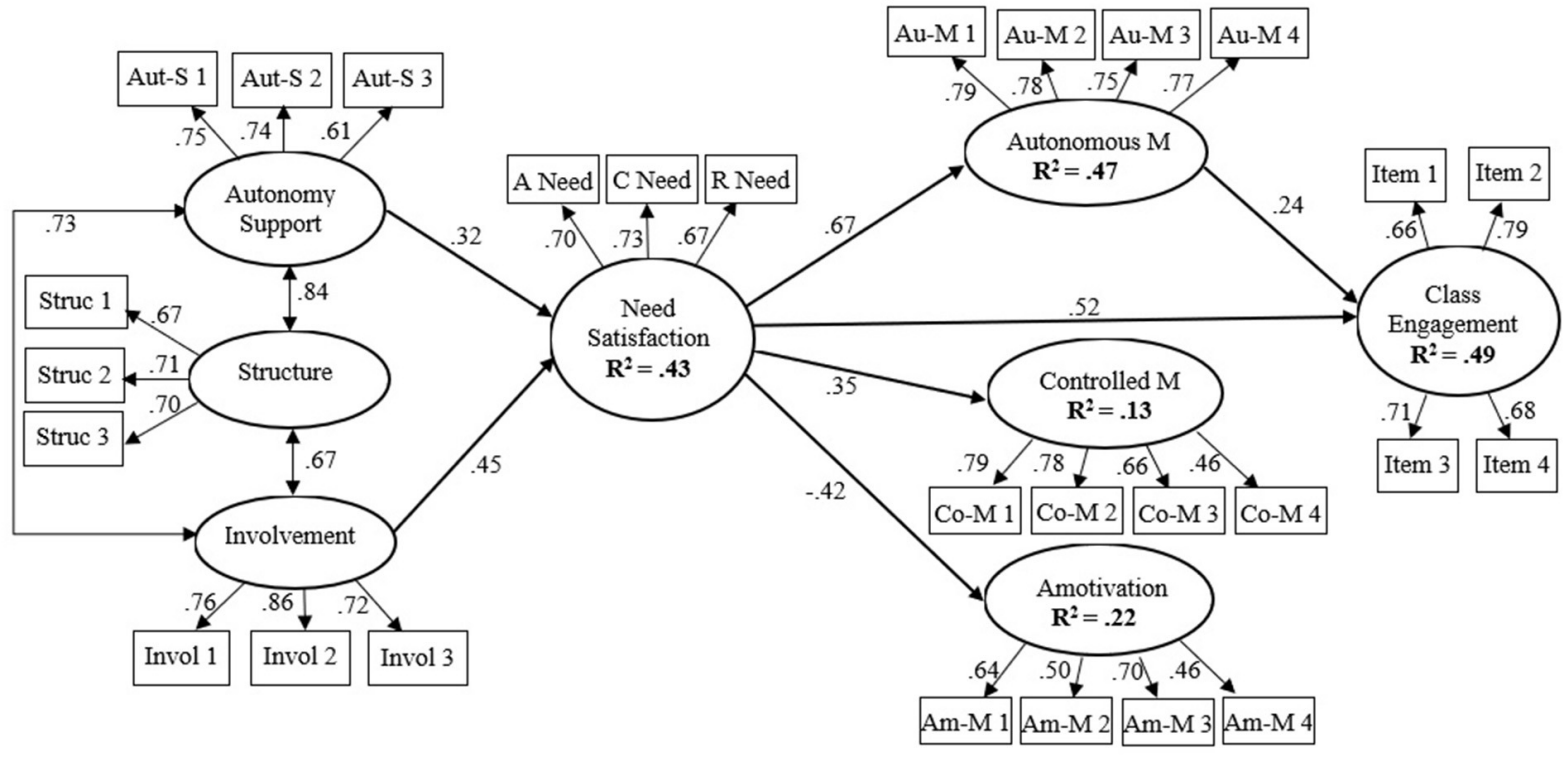
The structural model of the relations between teacher support, need satisfaction, motivation, and engagement among Chinese college students. All presented path coefficients are standardized and significant (*p* < 0.05). To avoid cluttering, this figure did not depict the non-significant path coefficients, the correlations between three subtypes of motivation, and the covariates (gender, grade, area, and major). Aut-S 1-3, Invol 1-3, and Struc 1-3 are the parcels for autonomy support, involvement, and structure, respectively. A Need, C Need, and R Need represented the needs for autonomy, competence, and relatedness, respectively. Autonomous M and Controlled M represented autonomous motivation and controlled motivation, respectively. Au-M 1-4, Co-M 1-4, and Am-M 1-4 are the parcels for autonomous motivation, controlled motivation, and amotivation, respectively. Gender, grade, family site, and major were not depicted for clarity, although they were controlled. The correlations of three teacher support dimensions and three motivation subtypes were as follows: *r*_autonomous motivation and controlled motivation_ = 0.43; *r*_autonomous motivation and amotivation_ = −0.35; *r*_controlled motivation and amotivation_ = 0.17.

As Figure 1 depicted (Model 1), both autonomy support and involvement were positively related to overall need satisfaction*, which* offered support for hypotheses 1a and 1b. However, different from hypothesis 1c, perceived structure could not statistically predict need satisfaction (β = −0.089, *p* = 0.519). Aligning with hypotheses 2a, 2b, *2c*, and 2d, need satisfaction was found to be positively linked to autonomous motivation, controlled motivation, and class engagement, whereas negatively related to amotivation. Considering the positive path from autonomous motivation to class engagement, hypothesis 3a was supported; however, SEM results failed to support hypotheses 3b and 3c, because controlled motivation (β = −0.102, *p* = 0.186) and amotivation (β = −0.005, *p* = 0.961) did not significantly relate to class engagement.

Table 4 presents the significant indirect effects of Model 1. In support of hypothesis 4a, need satisfaction played a statistically mediating role in the relations between two teacher support aspects (i.e., autonomy support and involvement) and three types of motivation (i.e., autonomous motivation, controlled motivation, and amotivation). However, only the chain of need satisfaction-autonomous motivation significantly mediated the association between perceived involvement and class engagement, supporting hypothesis 4b.

**Table 4:** The significant indirect path coefficients in model 1.

**Predictors**	**Mediators**	**Outcomes**	**β**	**SE**	***p***
**From teacher support to need to motivation**					
Involvement	Need	Autonomous motivation	0.30	0.06	<0.001
Autonomy support	//	//	0.22	0.09	0.023
Involvement	//	Controlled motivation	0.17	0.04	<0.001
Autonomy support	//	//	0.11	0.06	0.022
Involvement	//	Amotivation	−0.19	0.05	<0.001
Autonomy support	//	//	−0.14	0.07	0.043
**From teacher support to need to motivation to engagement**					
Sum of indirect from involvement to engagement			0.06	0.02	0.009
Involvement	Need-autonomous motivation	Engagement	0.07	0.03	0.015

## Discussion

SDT has asserted the effects of all three teacher support dimensions on motivational outcomes, however, most SDT-studies have only confirmed the contribution of teacher autonomy support or overall teacher support (Ryan and Deci, 2020). The overarching focus of the present study was to investigate a full sequential model which could help to test the joint effects of all three teacher support dimensions on student motivational outcomes. In general, after accounting for the nested nature of the data and four socio-demographic factors, the results of the present study were in agreement with most SDT hypotheses. That is, both perceived teachers' autonomy support and involvement were positively linked to students' need satisfaction, and the latter was positively associated with autonomous motivation, controlled motivation, and class engagement whereas negatively related to amotivation. However, only autonomous motivation was linked to class engagement. Finally, need satisfaction mediated the links from autonomy support and involvement to three motivational types, yet only the chain of need satisfaction-autonomous motivation statistically mediated the association between involvement support and class engagement.

### The SDT-motivation sequence among Chinese college students

Both autonomy support and involvement, but not structure, were found to contribute to Chinese college students' satisfaction of three basic psychological needs, which supported the findings in the research of Lietaert et al. (2015). However, these findings were not in line with the results of research, which has revealed that the influence of teachers' structure can be positive (Tucker et al., 2002; Taylor and Ntoumanis, 2007; De Naeghel et al., 2014), or conversely, negative (Hornstra et al., 2020; González-Peño et al., 2021). One reason for such inconsistent results is perhaps due to the various domains, that is, regarding the context of PE, reading, school activity, or classes in general. Another reason is related to the participants' characteristics. The samples of previous research included primary and high school students in the Western culture (e.g., Taylor and Ntoumanis, 2007), while our work was based on the experiences of Chinese college students. Compared with students in primary and high schools, university students have more opportunities to make choices in their learning programs, courses, and schedules (Ratelle et al., 2007). Indeed, in the present study, college students reported a higher level of teacher autonomy support but a lower level of structure, which might result in the feeling of incompetence and helplessness, and then yield the null relationship between perceived structure and need satisfaction.

As expected (hypotheses 2a, 2b, 2c, and 2d), the present model confirms that need satisfaction is a positive predictor of autonomous motivation, controlled motivation, and class engagement whereas is a negative predictor of amotivation. These results supported SDT-theoretical postulations (Ryan and Deci, 2020) and previous findings in PE class (e.g., Standage et al., 2005; Zamarripa et al., 2021). These findings support the essential importance of need satisfaction to promote student motivation and engagement.

Among the three types of motivation, only autonomous motivation was found to be a predictive factor of class engagement, which was contrary to our hypotheses 3b and 3c but in support of hypothesis 3a. The positive path from autonomous motivation to engagement replicated prior findings (e.g., Zhou et al., 2019b) and reconfirmed the generalizability of SDT postulates (Ryan and Deci, 2020). This finding reinforced that if students participated in the classes due to more self-determined reasons (enjoyment, pleasure, and importance of study), they would display higher levels of class engagement.

As alluded to earlier, the hypothesized paths from controlled motivation and amotivation to engagement-related outcomes tend to be inconsistent (Sánchez-Oliva et al., 2014). Aligned with prior studies (Behzadnia et al., 2018; Zamarripa et al., 2021; Leo et al., 2022), the current study found a non-significant link between controlled motivation and engagement. Notably, the bivariate correlation between these two variables was positive, but in the model, the path coefficient was non-significant. One reason for this result might be explained by the impacts of other antecedents on class engagement (Wu et al., 2014). For our data, when controlling for autonomous motivation, the partial correlation between controlled motivation and engagement was −0.014, which was not statistically significant (*p* = 0.710). The second reason might be related to the cross-sectional design, which was unable to obtain the maladaptive consequences of controlled motivation (Standage et al., 2005).

The path from amotivation to class engagement failed to reach statistical significance, which was not consistent with the SDT hypothesis. Amotivation has been shown to negatively predict engagement in PE class (e.g., Leo et al., 2022). However, this finding was supported by the prior PE research, which has reported the non-significant path from amotivation to emotional engagement (Standage et al., 2005; Sánchez-Oliva et al., 2014). Since Chinese traditional educational cultures stress teachers' authority and students' obedience (Zhou et al., 2019a), it is not surprising that Chinese college students may be accustomed to obeying their teachers' instructions and pretend to engage in classes even when they lack any intention in their learning activities.

Finally, our study highlights concern for the mediators of the relations between three teacher dimensions and class engagement. In line with SDT, need satisfaction was found to be a major mediator. Meanwhile, the chain of need satisfaction to autonomous motivation played a significant mediating role in the association between involvement and class engagement. This mediation chain expanded the results of previous research in the PE domain, concerning the relations between overall teacher support and class engagement (Leo et al., 2022), as well as between autonomy support and positive emotion during PE classes (Behzadnia et al., 2018). Martin (2009) has suggested that, as for class engagement, need satisfaction is likely to be a causative variable, whereas motivation appears to be more of a proximal variable.

### Practical implications, limitations, and future directions

To our knowledge, this is the first study to test simultaneously the combined contributions of all three teacher support dimensions to student motivational variables within an integrated model, which also extended previous SDT findings in a variety of aspects, including culture (Eastern context rather than Western context), domain (classes in general rather than PE class), and grade level (university rather than elementary and/or high school).

Despite the strengths, the present study has several limitations. The first is related to its cross-sectional design in terms of student self-reports. In the future, a longitudinal or experimental design should be conducted, which can provide causal support for our model. As for self-report, we cannot completely control for its common method bias or social desirability response bias, however, it is often the students' subjective experience of teacher support that is one of the strong predictors of motivational variables (Jang et al., 2010; Opdenakker, 2021). Hence, future research could conduct more objective assessments such as teachers' reports or observers' ratings, or assess social desirability bias as a control variable in the analysis.

The second concerns the sample. All the participants came from one university. The sample was predominantly female, although this was consistent with the gender rate in previous research among Chinese college students (Pan and Gauvain, 2012). Future studies with a sufficient sample size (e.g., 20 cases per parameter) with participants from distinct settings would assist in making our findings more generalized. Additionally, future research involving more classes is recommended to separately test the present model at the student and class levels.

The last limitation is about the motivational pathways. Our findings supported that autonomous motivation, controlled motivation, and engagement were predicted positively by students' perceived teacher support and need satisfaction, and they related negatively to amotivation. However, several SDT researchers in education have argued the bright and dark motivational pathways (Vansteenkiste and Ryan, 2013; Vansteenkiste et al., 2020). Specifically, autonomous motivation is primarily and positively predicted by need support context and need satisfaction, whereas controlled motivation and amotivation appear to be primarily and positively predicted by need-thwarting context and need frustration. Therefore, future research should consider the assessment of students' experience of need thwart and need frustration. Moreover, future research needs to expand to measure other aspects of the study variables in the pathway model, such as, need support provided by other social agents (e.g., friends and parents), integrated regulation, and different engagement-related variables (e.g., students' positive and negative emotions, concentration, and performance).

Taking into account the above weaknesses, our results provided tailor-made information about how teachers' supportive practices could foster student motivation and class engagement. Specifically, university teachers could help satisfy students' three psychological needs through autonomy-supportive and involved teaching behaviors, especially for students (freshmen and sophomores) who were exploring a relatively new environment (Amoura et al., 2015). In addition, given the lower levels of structure in our study, it is necessary for Chinese college teachers to create a well-structured learning context. Although perceived structure failed to predict need satisfaction in our model, SDT assumes that only when perceived structure with an abiding sense of autonomy support and involvement can teachers establish the optimal learning context (Ryan and Deci, 2020). Finally, the findings also provide specific implications for teachers' in-service training and university curriculum reform, such as taking into consideration training teachers' need supportive practices through a democratic instructional style (Burgueño et al., 2021).

## Data availability statement

The original contributions presented in the study are included in the article/supplementary material, further inquiries can be directed to the corresponding author/s.

## Ethics statement

The studies involving human participants were reviewed and approved by Zhejiang International Studies University, China. The patients/participants provided their written informed consent to participate in this study.

## Author contributions

LZ and XZ collected and analyzed the data. LZ drafted the manuscript. JH developed the manuscript. LZ, YG, and XT planned the study. All authors contributed to the article and approved the submitted version.

## Funding

This research was supported by Hangzhou Philosophy and Social Sciences Planning Project (Grant No. M22JC054) and Zhejiang Educational Sciences Planning Project (Grant No. 2022SCG401).

## Conflict of interest

The authors declare that the research was conducted in the absence of any commercial or financial relationships that could be construed as a potential conflict of interest.

## Publisher's note

All claims expressed in this article are solely those of the authors and do not necessarily represent those of their affiliated organizations, or those of the publisher, the editors and the reviewers. Any product that may be evaluated in this article, or claim that may be made by its manufacturer, is not guaranteed or endorsed by the publisher.

## Footnotes

^1^Based on the research of Olivier et al. (2021), we tested two alternative models, namely, the synergistic model and the bifactor-CFA global model. The results showed that our data did not support either of these two models because neither of them could converge.
